# Effects of different doses and preparations of zinc oxide in weanling piglets on performance, intestinal microbiota and microbial metabolites

**DOI:** 10.1093/tas/txaf073

**Published:** 2025-06-20

**Authors:** Jonathan Riedmüller, Alessandra Monteiro, Klaus Männer, Eva M Saliu, Wilfried Vahjen, Jürgen Zentek

**Affiliations:** Freie Universität Berlin, Königin-Luise-Straße 49, 14195 Berlin, Germany; Animine, 10 Rue Léon Rey Grange, 74960 Annecy, France; Freie Universität Berlin, Königin-Luise-Straße 49, 14195 Berlin, Germany; Freie Universität Berlin, Königin-Luise-Straße 49, 14195 Berlin, Germany; Freie Universität Berlin, Königin-Luise-Straße 49, 14195 Berlin, Germany; Freie Universität Berlin, Königin-Luise-Straße 49, 14195 Berlin, Germany

**Keywords:** microbiome, minerals, performance, piglets, zinc oxide

## Abstract

For years, high zinc levels (up to 3000 mg/kg feed) have been used to aid piglets during the weaning phase. However, studies revealed drawbacks like antimicrobial resistance and environmental impact. Since 2022, the EU limits zinc inclusion levels to 150 mg total zinc per kg. Therefore, alternative strategies for replacing high levels of zinc are of great interest. This study compares a potentiated zinc oxide source (HiZox^®^) and feed grade zinc oxide at various levels on piglet performance, fecal consistency and fecal microbiome.

The trial involved a total of 1,440 healthy weaned piglets (DanBred × Duroc; 50% barrows, 50% gilts; initial BW 10.1 ± 1.46 kg) over the 28-day duration of the experimental period. Piglets were randomly assigned to 12 treatment groups, each consisting of 12 pens, with 10 piglets per pen.

The trial comprised two periods: the starter period (days 1 to 14) focused on comparing the two zinc products, while all animals received a diet supplemented with 150 mg/kg of potentiated ZnO in the grower period (days 15 to 28). Treatment groups receiving feed grade ZnO were denoted as Z150, Z300, Z600, Z900, Z1500, and Z3000, while corresponding groups with the potentiated ZnO product were labeled H150, H300, H600, H900, H1500, and H3000, respectively. Body weight (BW) and feed intake (FI) were measured for every pen at days 1, 14 and 28. Fecal samples were collected on the same days.

During the starter phase (days 1 to 14), potentiated ZnO significantly improved average daily gain (ADG) compared to feed-grade ZnO (*P* ≤ 0.001), with the highest values observed at 3000 mg/kg (H3000: 247 g/d vs. Z3000: 233 g/d). Feed intake (FI) was also higher in potentiated ZnO groups (*P* ≤ 0.001), and feed conversion ratio (FCR) was more efficient (e.g., H3000: 1.2 vs. Z3000: 1.24; *P* = 0.001). In the grower phase residual effects from the starter phase persisted: animals previously fed potentiated ZnO had improved FCR (*P* = 0.003). Fecal microbiota analysis revealed that higher zinc levels reduced *Lactobacillus* abundance (P ≤ 0.001) and increased the presence of genera typical of adult pigs, such as *Clostridium* sensu stricto 1 and *Terrisporobacter* (P < 0.01). In conclusion, based on the observed shift in fecal microbiota composition characterized by a reduction in lactobacilli and an increase in proteobacteria due to heightened dietary zinc levels, it is advised to adjust zinc supplementation to 150 mg/kg after the initial 2 wk post-weaning.

## Teaser

Due to its significant relevance, this study has a major impact on the feeding strategy of weaned piglets, especially regarding the use of trace minerals like zinc oxide in feed. Moreover, this study provides further insights into the effects of different zinc oxide sources on both the animals and their microbiome.

## INTRODUCTION

Zinc is an essential trace mineral for animals, with a multitude of functions in the animal organism (Sloup et al., 2017). For a long time, zinc oxide (ZnO) has been widely used at pharmacological doses to effectively improve animal performance, reduce diarrhea in weaned piglets, increase feed intake and thus promote animal growth (Bonetti et al., 2021). Even before the ban of antimicrobial growth promoters (AGP) in the European Union (Regulation EC (1831/2003)) ZnO was found to be an alternative to AGP by stabilizing digestive health in the sensitive post-weaning period (Liu et al., 2018). Feeding high levels of ZnO to piglets showed similar results as the usage of AGP in regard of performance, fecal scores and microbial communities in the gut (Morales et al., 2012; Yu et al., 2017).

A large number of studies have investigated the effects and mechanisms of action of ZnO in the digestive tract of pigs. Initial work has shown that high doses of ZnO lead to profound effects on the intestinal microbiota (Højberg et al., 2005). Bacterial communities and their metabolic profiles have been shown to be altered with the inclusion of high levels of zinc in the feed of weaned piglets. Dietary zinc intake of 3000 mg/kg had a strong decreasing effect on lactic acid bacteria and increased the potential for enterobacteria to colonize the ileum of piglets (Vahjen et al., 2010). In addition to the effects on the intestinal microbiome, other associated effects on the digestive tract have been described. Several studies confirmed an increase of mucosal thickness, villus height and depth (Li et al., 2001; Zhu et al., 2017).

However, the continuous use of high levels of zinc in the feed of piglets has negative effects on the environment due to accumulation in manure and in soils. Furthermore, prevalence and spread of antibiotic resistant bacteria in feces is enhanced (Ciesinski et al., 2018). These problems are a threat for animals, humans and the environment. Therefore, in compliance with Directive 2001/82/EC, the marketing authorizations for veterinary medicinal products containing ZnO were effectively withdrawn in 2022. The maximum concentration of zinc used in the feed of piglets in Europe was limited to 150 mg/kg of zinc in complete feed for piglets in 2022. Efforts have been made to improve piglet management and sanitary conditions, together with dietary modifications. In this line, the development of alternative solutions for the weaning period became essential (Liu et al., 2018). Among the approaches to reduce the concentrations of dietary zinc for piglets the use of more efficient zinc sources is being explored. A study demonstrated that the physicochemical characteristics of ZnO sources could explain differences in their solubility and bioavailability (Cardoso, Roméo, et al., 2021). Studies comparing conventional zinc oxide with alternative zinc sources indicated that lower levels of the alternative zinc source used in the present study might be able to control the bacteria growth (Vahjen et al., 2012, 2016) and to improve intestinal integrity (Wang et al., 2019).

On this background, animal performance and fecal microbiome of post-weaning piglets were analyzed in this study to investigate dose dependent short-term effects of two zinc oxide sources with differing physicochemical properties. Furthermore, potential long-term impacts on performance were explored by continued feeding of ZnO at low concentrations.

## MATERIALS AND METHODS

### Ethical Statement

The trial was performed in accordance with the Animal Welfare Act of Germany approved by the local state office of health and social affairs (Landesamt für Gesundheit und Soziales, LaGeSo, no. A 0439/2017). Piglets used in the study were raised and treated according to European Union Directive 2010/63/EU  covering the protection of animals used for experimental or other purposes and according to the recommendation of Commission 2007/526/CE . Appropriate animal health and welfare inspections were carried out throughout the trial. After the study all animals were removed to the farm of origin.

### Study Design, Animals and Husbandry

A total number of 1,440 healthy weaned piglets (DanBred × Duroc; 50% barrows, 50% gilts: selected for uniformity with an initial body weight 10.1 ± 1.46 kg) were used in the 28-d trial. The piglets were weaned at 28 ± 2 d of age. Animals used for this trial were selected from a pool of approximately 1,800 piglets of both genders. The selection process focused on health and weight. The selected piglets were allotted equally according to body weight, litter, and gender to 144 pens (10 piglets per pen). We aimed for an initial average body weight of around 10 kg per pen.

Throughout the first week of the trial, the average barn temperature was set to 29°C in all stables. From the second week of the trial onward, the temperature was reduced by 2°C per week. Hence, at the end of the trial the temperature in the stables was 23°C. The relative humidity was within the range of 50% to 60%. The light regime comprised 16 h light and 8 h darkness.

### Diets and Feeding

Throughout the 4-week-trial, diets were offered ad libitum as mash (particle size range: 1.5 to 3 mm). Drinking water was freely available throughout the trial. The composition and calculated nutrient concentrations of the diets are listed in Table 1 (starter diets) and Table 2 (grower diets). Analyzed nutrients and mineral concentrations of iron, manganese, zinc and copper are listed in the Supplemental Table 1.

**Table 1.** Feed composition and calculated analysis of the experimental starter diets from d 1 to d 14 of the trial (as is)

Treatment groups		Z150	Z300	Z600	Z900	Z1500	Z3000	H150	H300	H600	H900	H1500	H3000
		**Ingredients**											
Wheat	%	25.390	25.390	25.390	25.390	25.390	25.390	25.390	25.390	25.390	25.390	25.390	25.390
Barley	%	25.000	25.000	25.000	25.000	25.000	25.000	25.000	25.000	25.000	25.000	25.000	25.000
Soybean meal (49% CP)	%	23.650	23.650	23.650	23.650	23.650	23.650	23.650	23.650	23.650	23.650	23.650	23.650
Rye	%	20.000	20.000	20.000	20.000	20.000	20.000	20.000	20.000	20.000	20.000	20.000	20.000
Limestone	%	1.650	1.650	1.650	1.650	1.650	1.650	1.650	1.650	1.650	1.650	1.650	1.650
Monocalcium phosphate	%	1.300	1.300	1.300	1.300	1.300	1.300	1.300	1.300	1.300	1.300	1.300	1.300
Mineral and vitamins ^1^^)^	%	1.200	1.200	1.200	1.200	1.200	1.200	1.200	1.200	1.200	1.200	1.200	1.200
L-Lysine HCL	%	0.580	0.580	0.580	0.580	0.580	0.580	0.580	0.580	0.580	0.580	0.580	0.580
Soybean oil	%	0.500	0.500	0.500	0.500	0.500	0.500	0.500	0.500	0.500	0.500	0.500	0.500
L-Threonine	%	0.200	0.200	0.200	0.200	0.200	0.200	0.200	0.200	0.200	0.200	0.200	0.200
DL-Methionine	%	0.190	0.190	0.190	0.190	0.190	0.190	0.190	0.190	0.190	0.190	0.190	0.190
L-Tryptophan	%	0.040	0.040	0.040	0.040	0.040	0.040	0.040	0.040	0.040	0.040	0.040	0.040
Tixosil ^2^^)^	%	0.285	0.270	0.240	0.210	0.150	0.000	0.286	0.271	0.242	0.214	0.156	0.013
ZnO (as is)	%	0.015	0.030	0.060	0.090	0.150	0.300						
HiZox (as is)	%							0.014	0.029	0.058	0.086	0.144	0.287
			**Calculated nutrient concentrations**										
AME ^3^^)^	MJ kg	13.10	13.10	13.10	13.10	13.10	13.10	13.10	13.10	13.10	13.10	13.10	13.10
Crude protein	%	19.80	19.80	19.80	19.80	19.80	19.80	19.80	19.80	19.80	19.80	19.80	19.80
Lysine	%	1.45	1.45	1.45	1.45	1.45	1.45	1.45	1.45	1.45	1.45	1.45	1.45
Methionine	%	0.49	0.49	0.49	0.49	0.49	0.49	0.49	0.49	0.49	0.49	0.49	0.49
Methionine + cystine	%	0.84	0.84	0.84	0.84	0.84	0.84	0.84	0.84	0.84	0.84	0.84	0.84
Threonine	%	0.92	0.92	0.92	0.92	0.92	0.92	0.92	0.92	0.92	0.92	0.92	0.92
Tryptophan	%	0.28	0.28	0.28	0.28	0.28	0.28	0.28	0.28	0.28	0.28	0.28	0.28
Crude fat	%	2.11	2.11	2.11	2.11	2.11	2.11	2.11	2.11	2.11	2.11	2.11	2.11
Crude fiber	%	3.05	3.05	3.05	3.05	3.05	3.05	3.05	3.05	3.05	3.05	3.05	3.05
Crude ash	%	5.88	5.88	5.88	5.88	5.88	5.88	5.88	5.88	5.88	5.88	5.88	5.88
Calcium	%	0.95	0.95	0.95	0.95	0.95	0.95	0.95	0.95	0.95	0.95	0.95	0.95
Phosphorus	%	0.68	0.68	0.68	0.68	0.68	0.68	0.68	0.68	0.68	0.68	0.68	0.68
Dig. Phosphorus	%	0.44	0.44	0.44	0.44	0.44	0.44	0.44	0.44	0.44	0.44	0.44	0.44
Sodium	%	0.18	0.18	0.18	0.18	0.18	0.18	0.18	0.18	0.18	0.18	0.18	0.18

^1^Contents per kg premix (without Zn): 600000 I.U. Vit. A (acetate); 120000 I.U. Vit. D_3_; 6000 mg Vit. E (α -tocopherol acetate); 200 mg Vit. K_3_ (MSB); 250 mg Vit. B_1_ (mononitrate); 420 mg Vit. B_2_ (cryst. riboflavin); 300 mg Vit. B_6_ (pyridoxin-HCl); 1500 µg Vit. B_12_; 3000 mg niacin (niacinamide); 12500 µg biotin (commercial, feed grade); 100 mg folic acid (cryst., commercial, feed grade); 1000 mg pantothenic acid (Ca d-pantothenate); 60000 mg choline (chloride); 5000 mg iron (iron carbonate); 6000 mg manganese manganese oxide); 1000 mg copper (copper oxide); 45 mg iodine (calcium-iodate); 20 mg selenium (sodium-selenite); 140 g sodium (NaCl); 55 g magnesium (magnesium sulfate); carrier: calcium carbonate (calcium min 38%); 0.25 g ß-apo-carotinoide acid-ester; 0.33 g canthaxanthin.

^2^> 97% silicon dioxide.

^3^Calculated by using the estimation given by DLG 2013.

**Table 2.** Feed composition and calculated analysis of the experimental grower diets from d 15 to d 28 of the trial (as is)

Treatment groups		Z150	Z300	Z600	Z900	Z1500	Z3000	H150	H300	H600	H900	H1500	H3000
		**Ingredients**											
Barley	%	27.585	27.585	27.585	27.585	27.585	27.585	27.585	27.585	27.585	27.585	27.585	27.585
Wheat	%	25.690	25.690	25.690	25.690	25.690	25.690	25.690	25.690	25.690	25.690	25.690	25.690
Soybean meal (49% CP)	%	21.580	21.580	21.580	21.580	21.580	21.580	21.580	21.580	21.580	21.580	21.580	21.580
Rye	%	20.000	20.000	20.000	20.000	20.000	20.000	20.000	20.000	20.000	20.000	20.000	20.000
Limestone	%	1.520	1.520	1.520	1.520	1.520	1.520	1.520	1.520	1.520	1.520	1.520	1.520
Minerals & vitamins ^1^^)^	%	1.200	1.200	1.200	1.200	1.200	1.200	1.200	1.200	1.200	1.200	1.200	1.200
Monocalcium phosphate	%	1.180	1.180	1.180	1.180	1.180	1.180	1.180	1.180	1.180	1.180	1.180	1.180
Soybean oil	%	0.500	0.500	0.500	0.500	0.500	0.500	0.500	0.500	0.500	0.500	0.500	0.500
L-Lysine HCL	%	0.460	0.460	0.460	0.460	0.460	0.460	0.460	0.460	0.460	0.460	0.460	0.460
L Threonine	%	0.130	0.130	0.130	0.130	0.130	0.130	0.130	0.130	0.130	0.130	0.130	0.130
DL-Methionine	%	0.120	0.120	0.120	0.120	0.120	0.120	0.120	0.120	0.120	0.120	0.120	0.120
L-Tryptophan	%	0.020	0.020	0.020	0.020	0.020	0.020	0.020	0.020	0.020	0.020	0.020	0.020
HiZox (as is)	%	0.015	0.015	0.015	0.015	0.015	0.015	0.015	0.015	0.015	0.015	0.015	0.015
		**Calculated nutrient concentrations**											
AME ^2^^)^	MJ kg	12.20	12.20	12.20	12.20	12.20	12.20	12.20	12.20	12.20	12.20	12.20	12.20
Crude protein	%	18.90	18.90	18.90	18.90	18.90	18.90	18.90	18.90	18.90	18.90	18.90	18.90
Lysine	%	1.30	1.30	1.30	1.30	1.30	1.30	1.30	1.30	1.30	1.30	1.30	1.30
Methionine	%	0.41	0.41	0.41	0.41	0.41	0.41	0.41	0.41	0.41	0.41	0.41	0.41
Methionine + cystine	%	0.75	0.75	0.75	0.75	0.75	0.75	0.75	0.75	0.75	0.75	0.75	0.75
Threonine	%	0.82	0.82	0.82	0.82	0.82	0.82	0.82	0.82	0.82	0.82	0.82	0.82
Tryptophan	%	0.25	0.25	0.25	0.25	0.25	0.25	0.25	0.25	0.25	0.25	0.25	0.25
Crude fat	%	2.12	2.12	2.12	2.12	2.12	2.12	2.12	2.12	2.12	2.12	2.12	2.12
Crude fiber	%	3.09	3.09	3.09	3.09	3.09	3.09	3.09	3.09	3.09	3.09	3.09	3.09
Crude ash	%	5.56	5.56	5.56	5.56	5.56	5.56	5.56	5.56	5.56	5.56	5.56	5.56
Calcium	%	0.88	0.88	0.88	0.88	0.88	0.88	0.88	0.88	0.88	0.88	0.88	0.88
Phosphorus	%	0.64	0.64	0.64	0.64	0.64	0.64	0.64	0.64	0.64	0.64	0.64	0.64
Dig. Phosphorus	%	0.42	0.42	0.42	0.42	0.42	0.42	0.42	0.42	0.42	0.42	0.42	0.42
Sodium	%	0.18	0.18	0.18	0.18	0.18	0.18	0.18	0.18	0.18	0.18	0.18	0.18

^1^Contents per kg premix (without Zn): 600000 I.U. Vit. A (acetate); 120000 I.U. Vit. D_3_; 6000 mg Vit. E (α -tocopherol acetate); 200 mg Vit. K_3_ (MSB); 250 mg Vit. B_1_ (mononitrate); 420 mg Vit. B_2_ (cryst. riboflavin); 300 mg Vit. B_6_ (pyridoxin-HCl); 1500 µg Vit. B_12_; 3000 mg niacin (niacinamide); 12500 µg biotin (commercial, feed grade); 100 mg folic acid (cryst., commercial, feed grade); 1000 mg pantothenic acid (Ca d-pantothenate); 60000 mg choline (chloride); 5000 mg iron (iron carbonate); 6000 mg manganese manganese oxide); 1000 mg copper (copper oxide); 45 mg iodine (calcium-iodate); 20 mg selenium (sodium-selenite); 140 g sodium (NaCl); 55 g magnesium (magnesium sulfate); carrier: calcium carbonate (calcium min 38%); 0.25 g ß-apo-carotinoide acid-ester; 0.33 g canthaxanthin.

^2^Calculated by using the estimation given by DLG 2013.

The treatments comprised six inclusion levels (150, 300, 600, 900, 1500, 3000 mg/kg Zn) of two different ZnO products: a conventional feed-grade ZnO (ZnO Maximo; Zinc Nacional, Mexico) and a potentiated ZnO (HiZox^®^, Animine, France). The differences in chemical composition and physicochemical characteristics of both sources, as measured in a former study (Cardoso, Narcy, et al., 2021), are presented in the Supplemental Table 2.

Animals were allotted to 12 groups, with 12 pens per group and 10 piglets per pen. Pens were treated as experimental unit for performance data. The trial was divided into two periods of 14 d each: in the starter period (1 to 14d) the two zinc products were compared (period 1), while all animals received the same diet supplemented with 150 mg/kg of potentiated ZnO in the grower period (period 2). Treatment groups that included the feed-grade ZnO were labeled as Z150, Z300, Z600, Z900, Z1500 and Z3000, while corresponding treatment groups with potentiated ZnO product were labeled H150, H300, H600, H900, H1500 and H3000, respectively. All diets met or slightly exceeded the recommendations for weaned piglets (German Society of Nutrition Physiology, 2006) with the exception of zinc.

### Performance

Body weight (BW) and feed intake (FI) were determined for every pen on days 1, 14 and 28. Measurements were then used to calculate the average daily gain (ADG) and feed conversion ratio (FCR). Calculations were done for the two experimental periods as well as for the overall experimental period (d 1—d 28 on trial).

### Fecal Score

Throughout the trial, occurrence and severity of post-weaning diarrhea was assessed daily. A modified version of the diarrhea scale used by Liu et al was used to describe the fecal consistency (Liu et al., 2010). The scale reached from 0 to 3, with 0 representing the highest and 3 representing the lowest fecal firmness level (0 = well-formed feces, firm to cut; 1 = pasty feces without falling out of shape; 2 = pasty feces falling out of shape upon contact with surfaces; 3 = liquid diarrhea). After daily monitoring of each pen, a diarrhea score was calculated per pen. The assessment of the score was done by two trained persons, who had no knowledge of the dietary treatment allotment throughout the whole experimental period. For the respective feeding periods, daily scores of each pen were added up and then divided by the number of days of that period to calculate the daily diarrhea score of the pen per feeding period.

### Sampling of Feces

Fresh fecal samples of all 144 pens were collected at d 14 on trial. Samples of treatment groups Z150, Z600, Z1500, Z3000, H150, H600, H1500 and H3000 were used for the analysis of bacterial metabolites, quantitative PCR determination of *Escherichia*/*Hafnia*/*Shigella* and selected pathogenic factors (*estIb*, *estII, FedA*, *fae*) as well as for 16S rDNA sequencing of the fecal microbiome.

### Fecal Analysis

#### Microbial metabolites.

Short chain fatty acids concentrations were measured using gas chromatography (Agilent Technologies 6890N, autosampler G2614A, and injection tower G2613A; Network GC Systems, Germany) as described elsewhere (Kröger et al., 2017). Caproic acid was used as an internal standard. Analysis of D- and L-lactate was done using high performance liquid chromatography (HPLC; Agilent 1100; Agilent Technologies, Germany).

#### Extraction of fecal DNA.

A commercial extraction kit was used for the extraction of fecal DNA (QIAamp® PowerFaecal® Pro DNA Kit; Qiagen, Germany). Samples were homogenized with a FastPrep-24TM 5G (M.P. Biomedicals LLC, USA). The extraction was carried out according to the manufacturer’s instructions with an additional lysis step (65°C, 10 min). DNA extracts were stored at -30 °C until further analysis. DNA yield was determined with fluorescent detection (QuantiFluor® dsDNA System; Promega, Germany).

#### Quantitative PCR.

DNA extracts were diluted with water to equimolar concentrations before amplification. A commercial master mix (Brilliant II SYBR® Green QPCR Master Mix with Low ROX; Stratagene, Amsterdam, Netherlands) was used to amplify the 16S rDNA gene of the group *Escherichia*/*Hafnia*/*Shigella* and selected pathogenic factors (*E. coli* toxins Ib, II, *estIb* and *estII, FedA*, *fae)* on a qPCR cycler (AriaMx real-time PCR system, Agilent, USA). Primer and PCR conditions are given in Supplemental Table 3.

#### 16S rDNA Sequencing.

16S-rDNA sequencing was performed by LGC Genomics GmbH (Berlin, Germany), and the obtained sequencing data were analyzed as described elsewhere (Grześkowiak et al., 2019). In short, the 341F-785R V3-V4 region of the 16S rDNA was targeted and sequenced. After demultiplexing, combined reads were analyzed using the QIIME2 pipeline and the DADA2 routine within QIIME2, excluding reads with less than five sequences. After quality control and rearification, amplicon sequence variants were taxonomically assigned via the SILVA SSU database.

### Statistical Analyses

A priori power analysis was conducted using a fixed-effects ANOVA model with 12 treatment groups and a total sample size of 144 observations (12 pens per treatment group). The primary outcome was body weight. Assuming a medium effect size (Cohen’s *f* = 0.4), the design achieved a statistical power of 90.4% at an alpha level of 0.05.

Pens were used as the experimental unit for all performance-related outcomes, with treatments randomly assigned at the pen level. Consequently, pen effects were not included in the model as fixed effects. This approach is consistent with the experimental design, where each pen represented an independent observational unit.

The statistical program “R” was used to perform a two factorial analysis of variance for each measured parameter. Source and concentration of zinc were used as the two factors, *P*-values are given for each variable as well as for the interaction of the two factors, values ≤ 0.05 were considered statistically significant. The post-hoc comparison was performed by the Tukey-HSD test. Significant differences are given in the tables by different superscripts.

Comparative analysis of the fecal microbiome was done via the Linear Discriminant Analysis for Effect Size (LEfSe), which combines linear discriminant analysis with statistical tests to estimate the significant effect size of each feature (Segata et al., 2011).

## RESULTS

### Animal Performance

The influence of zinc inclusion level and zinc source on performance of the animals is displayed in Table 3. During period 1 of this trial, ADG was higher with increasing dietary zinc concentrations (*P ≤* 0.001). Compared to piglets fed the feed-grade ZnO, the potentiated ZnO product showed a significantly higher ADG. FI during period 1 was influenced by both the dosage of zinc (*P* = 0.001) and zinc source (*P ≤* 0.001). Piglets receiving the potentiated ZnO product displayed higher FI compared to animals fed diets with feed-grade ZnO. Significant differences between the groups could be seen between H3000 and groups Z150, Z300, Z600. FCR improved with increasing inclusion levels of ZnO (*P ≤ *0.001) and piglets fed the potentiated ZnO product showed a better FCR than piglets fed the feed-grade zinc source (*P* = 0.001). There were no significant effects on BW at the end of period 1.

**Table 3.** Effect of two different dietary sources of zinc oxide (feed-grade ZnO and a potentiated ZnO product) on zootechnical performance of weaned piglets

														*P* - value^2^		
	Z150	Z300	Z600	Z900	Z1500	Z3000	H150	H300	H600	H900	H1500	H3000	SEM^1^	C	S	CxS
Body weight, kg																
d 1	10.1	10.1	10.1	10.1	10.1	10.1	10.1	10.1	10.0	10.1	10.1	10.1	0.003	1.000	0.966	1.000
d 14	12.5	12.5	12.5	12.9	13.2	13.3	12.8	13.1	13.2	13.3	13.4	13.5	0.103	0.498	0.130	0.993
d 28	19.6	19.5	19.8	19.9	20.1	20.3	19.5	19.9	20.1	20.2	20.4	20.7	0.105	0.349	0.312	0.997
D 1 to 14 (period 1)																
ADG, g	177	173	177	201	222	233	199	217	223	230	238	247	7.393	≤ 0.001	≤ 0.001	0.289
ADFI, g	239	234	234	257	277	288	261	275	279	279	284	295	6.201	0.001	≤ 0.001	0.389
FCR	1.35	1.35	1.33	1.28	1.24	1.24	1.32	1.27	1.25	1.21	1.19	1.20	0.016	≤ 0.001	0.001	0.905
D 15 to 28 (period 2)																
ADG, g	502	503	516	505	498	497	478	487	498	497	499	510	2.806	0.613	0.127	0.450
ADFI, g	727	737	746	726	717	696	691	702	709	702	694	686	5.570	0.172	0.001	0.914
FCR	1.45	1.47	1.45	1.44	1.44	1.40	1.44	1.44	1.43	1.42	1.39	1.35	0.009	≤ 0.001	0.003	0.646
D 1 to 28 (Overall)																
ADG, g	339	338	346	353	360	365	339	352	360	363	369	378	3.743	≤ 0.001	0.009	0.856
ADFI, g	483	486	490	491	497	492	476	488	494	491	489	491	1.588	0.724	0.752	0.985
FCR	1.42	1.44	1.41	1.39	1.38	1.35	1.41	1.39	1.37	1.35	1.33	1.30	0.012	≤ 0.001	≤ 0.001	0.754

^1^Values are presented as means and standard error of the means (SEM).

^2^Statistical analysis was performed using two-factor ANOVA (zinc source × zinc concentration). The pen (n = 12 pens per treatment, 10 piglets per pen) served as the experimental unit. Differences were considered statistically significant at P ≤ 0.05.

In period 2 of the trial, where all piglets were fed 150 mg/kg of the potentiated ZnO product, FCR was significantly affected by the ZnO-concentration and -source, that were fed during period 1. Piglets that formerly received feed-grade ZnO showed a higher FCR compared to groups that already received the potentiated ZnO product during period 1 (*P =* 0.001). This is in part due to a significantly lower ADFI seen for animals fed the potentiated ZnO product before. The lowest FCR was found in the group receiving the highest concentration of the potentiated ZnO product, with significant differences to groups Z150 to Z1500 and groups H150 to H600.

For the overall trial, a clear effect on ZnO-concentration and -source was found for ADG and FCR. Piglets that received the potentiated ZnO product showed a significantly higher ADG (*P* = 0.009) compared to piglets that received feed-grade ZnO. Effects on FCR could also be seen for both source and dose (*P *≤ 0.001). The potentiated ZnO product showed a better FCR than piglets fed feed-grade ZnO. No statistical differences were recorded for ADFI.

### Fecal Consistency

Both zinc-concentration and -source showed significant interaction with fecal consistency (Table 4). During the first period, increasing zinc concentration increased fecal firmness, while zinc source showed a trend towards increased firmness in animals fed the potentiated ZnO product. After removal of high dietary zinc in period 2 of the trial, the tendency for increased fecal firmness remained in aninals previously fed higher dietary zinc concentrations. The same effect was observed for the potentiated ZnO product compared to the feed-grade ZnO product.

**Table 4.** Effect of two different sources of zinc oxide (feed-grade ZnO and the potentiated ZnO product) on fecal consistency of weaned piglets

														*P* - value^2^		
	Z150	Z300	Z600	Z900	Z1500	Z3000	H150	H300	H600	H900	H1500	H3000	SEM^1^	C	S	CxS
Fecal consistency																
D 1 to 14 (period 1)	0.9	0.7	0.7	0.8	0.8	0.5	0.7	0.8	0.7	0.6	0.6	0.5	0.038	0.019	0.074	0.127
D 15 to 28 (period 2)	0.4	0.4	0.3	0.3	0.2	0.2	0.3	0.3	0.2	0.2	0.1	0.1	0.027	0.004	0.013	0.992
D 1 to 28 (Overall)	0.6	0.5	0.5	0.6	0.5	0.3	0.5	0.6	0.5	0.4	0.4	0.3	0.031	≤ 0.001	0.003	0.234

Values represent the average fecal scores of each treatment group for each feeding period respectively. A scale was used that reached from 0 to 3, with 0 representing the highest and 3 representing the lowest fecal firmness level (0 = well-formed feces, firm to cut; 1 = pasty feces without falling out of shape; 2 = pasty feces falling out of shape upon contact with surfaces; 3 = liquid diarrhea).

^1^Values are presented as means and standard error of the means (SEM).

^2^Statistical analysis was performed using two-factor ANOVA (zinc source × zinc concentration). The pen (n = 12 pens per treatment, 10 piglets per pen) served as the experimental unit. Differences were considered statistically significant at P ≤ 0.05. Different superscript letters indicate significant differences between treatment means (Tukey-HSD test).

### Fecal Microbiome

#### Fecal microbiome composition.

Zinc concentration significantly modified the bacterial composition for *Firmicutes*, *Actinobacteria* and *Bacteroides*, but zinc source was not a factor (Supplemental Figure 1). The major genera for the dominating *Firmicutes* were *Lactobacillus* with 20% to 66% abundance, followed by *Clostridium* Sensu Stricto 1 (7% to 30%), *Streptococcus* (0.2% to 12%), and *Terrisporobacter* (0.8% to 5%) (see Table 5).

**Table 5:** Effect of two different dietary sources of zinc oxide (feed-grade ZnO and a potentiated ZnO product) on dominant bacterial genera in the feces of weaned piglets on day 14 of the trial

										P-value^1^	
	Z150	Z600	Z1500	Z3000	H150	H600	H1500	H3000	C	S	CxS
Lactobacillus	44.84 ± 14.96	65.99 ± 9.55	36.25 ± 20.83	31.71 ± 17.12	42.35 ± 11.04	50.31 ± 15.83	18.16 ± 13.15	20.49 ± 16.30	< 0.001	< 0.001	0.306
Clostridium_sensu_stricto_1	14.43 ± 8.79	7.24 ± 6.73	14.85 ± 17.01	25.31 ± 10.07	18.02 ± 17.77	20.97 ± 15.16	30.36 ± 16.56	20.80 ± 13.96	0.060	0.014	0.048
Streptococcus	1.48 ± 1.68	0.17 ± 0.15	1.9 ± 1.92	4.49 ± 4.99	2.97 ± 4.92	1.27 ± 2.41	4.82 ± 10.39	11.88 ± 15.2	0.003	0.028	0.397
Terrisporobacter	2.59 ± 1.86	0.80 ± 0.66	2.26 ± 2.18	4.02 ± 1.89	2.17 ± 1.95	3.32 ± 2.10	5.04 ± 2.34	4.42 ± 3.69	0.003	0.005	0.033
Subdoligranulum	4.02 ± 3.13	2.38 ± 1.98	2.82 ± 2.07	1.51 ± 0.93	3.25 ± 2.67	0.84 ± 0.59	5.77 ± 4.15	4.00 ± 2.39	0.002	0.124	0.003
Bifidobacterium	2.43 ± 3.13	1.68 ± 2.65	1.44 ± 1.49	5.39 ± 4.87	2.76 ± 3.47	1.87 ± 1.81	0.60 ± 0.63	4.16 ± 4.25	< 0.001	0.541	0.775
Blautia	3.07 ± 1.18	3.27 ± 1.50	4.25 ± 2.38	2.42 ± 1.30	3.64 ± 2.18	1.96 ± 1.44	4.01 ± 2.49	4.65 ± 2.33	0.061	0.429	0.016
Coprococcus_3	1.07 ± 0.49	0.80 ± 0.61	1.97 ± 1.63	0.62 ± 0.24	0.92 ± 0.47	0.94 ± 0.61	1.03 ± 0.68	0.97 ± 0.67	0.01	0.347	0.028
Agathobacter	1.33 ± 1.04	1.73 ± 0.89	2.23 ± 1.75	2.59 ± 2.59	2.75 ± 2.27	1.01 ± 0.64	1.69 ± 0.88	2.97 ± 1.82	0.034	0.678	0.095
unknown_Family_Lachnospiraceae	3.75 ± 1.89	3.37 ± 1.32	5.1 ± 2.43	3.02 ± 1.25	4.09 ± 1.89	2.55 ± 1.18	4.05 ± 1.83	3.48 ± 1.23	0.006	0.436	0.281
Coprococcus_1	0.45 ± 0.19	0.72 ± 0.44	0.46 ± 0.39	0.35 ± 0.28	0.41 ± 0.30	0.60 ± 0.84	0.36 ± 0.37	1.05 ± 1.01	0.141	0.318	0.032
Dorea	2.22 ± 1.62	1.33 ± 0.72	1.20 ± 0.84	0.85 ± 0.51	1.28 ± 0.93	0.79 ± 0.51	1.16 ± 0.63	1.91 ± 0.90	0.047	0.531	0.001
unknown_Order_Lactobacillales	0.65 ± 0.34	0.52 ± 0.34	0.41 ± 0.09	0.87 ± 0.19	0.66 ± 0.35	0.83 ± 0.35	0.59 ± 0.23	0.67 ± 0.14	0.010	0.200	0.011
Faecalibacterium	0.46 ± 0.35	0.32 ± 0.21	0.88 ± 0.77	0.53 ± 0.84	0.64 ± 0.37	0.17 ± 0.17	1.26 ± 1.12	2.46 ± 1.77	< 0.001	0.001	< 0.001
Ruminococcaceae_UCG_008	0.64 ± 0.51	0.39 ± 0.33	0.90 ± 0.44	0.29 ± 0.13	0.58 ± 0.69	0.46 ± 0.34	0.76 ± 0.55	0.36 ± 0.17	0.001	0.887	0.776
unknown_Family_Clostridiaceae_1	0.36 ± 0.20	0.38 ± 0.25	0.24 ± 0.06	0.43 ± 0.11	0.39 ± 0.19	0.51 ± 0.24	0.35 ± 0.21	0.26 ± 0.17	0.049	0.551	0.023
unknown_Family_Muribaculaceae	2.33 ± 2.15	0.50 ± 0.59	2.01 ± 2.18	1.08 ± 0.83	1.07 ± 0.99	0.87 ± 0.57	3.13 ± 2.04	1.26 ± 2.35	0.001	0.759	0.092
Marvinbryantia	0.96 ± 0.44	0.44 ± 0.14	1.06 ± 0.46	0.82 ± 0.39	0.72 ± 0.33	0.58 ± 0.26	0.69 ± 0.50	0.92 ± 0.37	0.002	0.239	0.046
Lachnospira	0.25 ± 0.18	0.12 ± 0.07	0.60 ± 0.43	0.40 ± 0.38	0.46 ± 0.45	0.29 ± 0.15	0.40 ± 0.33	0.31 ± 0.23	0.012	0.784	0.063
Ruminococcaceae_UCG_002	1.43 ± 1.61	0.11 ± 0.10	1.05 ± 0.80	0.31 ± 0.31	0.34 ± 0.25	0.40 ± 0.29	0.77 ± 0.60	0.26 ± 0.23	< 0.001	0.049	0.008

^1^Statistical analysis was performed using two-factor ANOVA (zinc source × zinc concentration). The pen (n = 12 pens per treatment, 10 piglets per pen) served as the experimental unit. Differences were considered statistically significant at P ≤ 0.05. Different superscript letters indicate significant differences between treatment means (Tukey-HSD test).

For these dominating genera not only zinc concentration, but also zinc source was a factor. Thus, *Lactobacillus* showed a stronger declince for the potentiated zinc product than for the feed-grade ZnO. Furthermore, the streptococci gained a higher abundance in animals fed the potentiated zinc product. The potentiated zinc product generally led to a higher abundance of bacterial genera that are typically dominant in adult pigs (*Clostridium* sensu stricto 1, *Terrisporobacter*, *Faecalibacterium*).

#### Bacterial Metabolites.

Fecal concentrations of short chain fatty acids (SCFA) and L- and D-Lactic acid were measured at the end of period 1 (Supplemental Table 4). No significant effects on zinc-source or -concentration were observed for SCFA, except for isovaleric acid. Overall, a trend for higher total SCFA was detected in fecal samples from animals fed the potentiated ZnO product. Increased supplementation of both ZnO products showed a repressing effect for fecal lactate concentrations (*P *≤ 0.001). Furthermore, the ratio of L- to D- lactate as indicator for bacterial metabolism was affected by both concentration and ZnO-source (*P *≤ 0.001). Compared to the feed-grade ZnO, the potentiated zinc source led to higher L/D-lactate ratios.

#### Discriminative analysis of the fecal microbiota.

16S abundance data was used to identify differential features in the fecal microbiota using the Linear Discriminant Analysis Effect Size (LEfSe) method. This approach contrasts relative abundances to find features that most likely explain intergroup differences.

**Figure 1.**
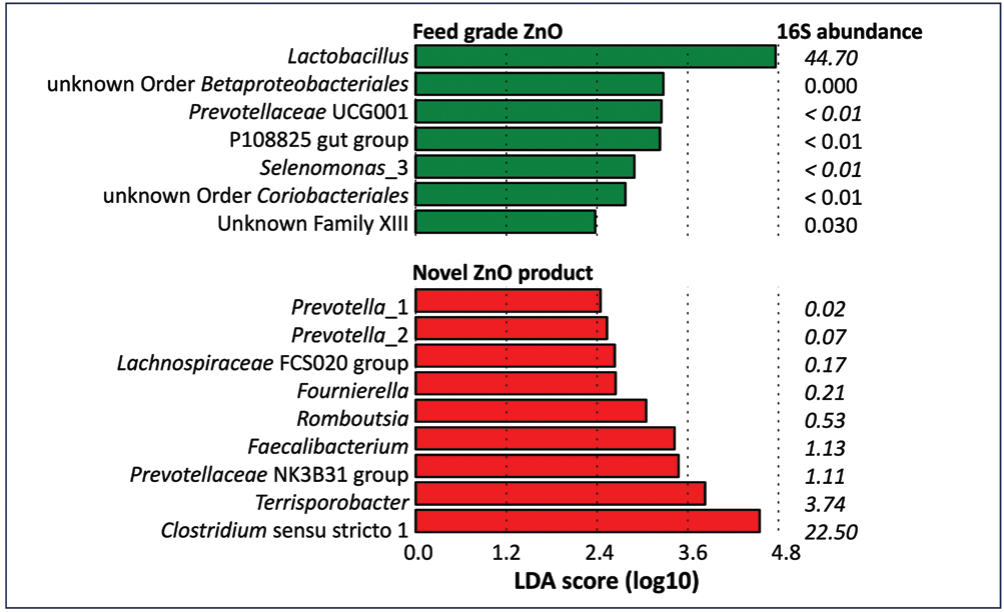
Comparative analysis of the fecal microbiome via Linear Discriminant Analysis for Effect Size (LEfSe).

Figure 1 shows the analysis of combined data from all treatment groups, separated by zinc source (n = 46 per group). For feed-grade ZnO fed animals, the most pronounced group difference was assorted to the lactobacilli. Compared to the actual abundance, all other genera played only a minor role. However, the most prominent group differences in animals fed the potentiated zinc product were found for more dominant genera (> 0.1 %). Features that are most probable to modify the microbiota in the potentiated zinc fed animals belong to bacteria found in adult animals (*Clostridium* sensu stricto 1, *Terrisporobacter*, *Faecalibacterium*).

### Fecal Escherichia spp., Enterotoxins and Fimbrial Genes

The quantitative determination of *Escherichia*/*Hafnia*/*Shigella* (*Escherichia* spp.) and relevant pathogenic factors are shown in Table 6. Significant effects of zinc-source and -concentration were observed for the concentration of *Escherichia* spp. The feed-grade ZnO showed the highest *Escherichia* spp. copy numbers at 1500 mg/kg, while the potentiated ZnO product led to highest copy numbers of *Escherichia* spp. in the 600 mg/kg group. A similar course was found for *E. coli* pathogenic factors. The *E. coli* toxin *estII* showed a gradual decline in feed-grade treatment groups, while the potentiated ZnO product led to highest copy numbers in the 600 mg/kg group with a drastic decrease at the highest ZnO concentration. No noteworthy changes were found for the analyzed *E. coli* fimbriae. However, both ZnO-products led to a sharp increase for *fae* and *FedA* in the respective highest concentrations.

**Table 6.** Effect of two different dietary sources of zinc oxide (feed-grade ZnO and a potentiated ZnO product) on fecal Enterobacteria and enterotoxins [log_10_(copy numbers/g feces)] in weaned piglets on day 14 of the trial

											*P* - value^2^	
	Z150	Z600	Z1500	Z3000	H150	H600	H1500	H3000	SEM^1^	C	S	CxS
Fecal bacterial Group												
*Escherichia/Hafnia/Shigella*	8.14	8.20	8.66	8.24	8.66	9.29	8.59	8.14	0.140	0.058	0.020	0.020
*E.coli* enterotoxins												
* estIb*	6.43	6.56	6.11	6.19	6.60	7.34	7.14	5.76	0.186	0.048	0.116	0.153
* estII*	6.72	6.57	6.47	6.16	6.81	8.00	7.05	5.41	0.262	0.002	0.213	0.039
Fimbrial genes												
* Fae*	2.44	1.98	0.84	1.68	2.91	1.78	0.73	3.83	0.365	0.013	0.220	0.258
* FedA*	5.21	5.17	4.95	6.80	6.23	5.69	5.68	6.94	0.266	0.030	0.136	0.886

^1^Values are presented as means and standard error of the means (SEM).

^2^Statistical analysis was performed using two-factor ANOVA (zinc source × zinc concentration). The pen (n = 12 pens per treatment, 10 piglets per pen) served as the experimental unit. Differences were considered statistically significant at P ≤ 0.05. Different superscript letters indicate significant differences between treatment means (Tukey-HSD test).

## DISCUSSION

The aim of this study was to explore the impact of varying dietary zinc inclusion levels on performance and fecal microbiota in weaned piglets. Our investigation aimed at exploring the connection between zinc levels and crucial parameters to minimize zinc consumption while preserving its advantages. Furthermore, we conducted a comparison between conventional feed-grade zinc oxide and its potentiated variant, analyzing their impacts on animal performance, fecal consistency, and the composition of the microbiome. Our premise was that the potentiated ZnO, due to unique physicochemical properties, could be effective at lower doses, maintaining its beneficial impact. Furthermore, the study aimed to determine whether varying zinc concentrations, typically administered for 2 wk post-weaning, could have lasting effects on performance even after reducing zinc levels to 150 mg/kg for an additional 2 wk.

### Performance.

This study supports previous findings of growth promoting characteristics of high levels of zinc used in the feed of weaned piglets. Although performance variables were measured at multiple time points (days 1, 14, and 28), the dataset structure was organized around treatment group averages per time point rather than individual longitudinal records. Therefore, we performed time point-specific analyses rather than employing a repeated-measures mixed model. We acknowledge this as a limitation, as mixed-effects models can more effectively account for within-subject correlations over time. Nonetheless, given the available data structure, this approach allowed for consistent comparisons of treatment effects across defined experimental phases. As expected, ADG, ADFI and FCR significantly improved with increasing levels of zinc in the first period of the trial. This effect has been observed in similar studies on dietary ZnO in weaned piglets (Cho et al., 2015; Dębski, 2016). However, both ZnO products failed to improve body weight at the end of the 14d trial phase (period 1), most probably due to the relatively high starting weight of the animals (10.1 kg). This relatively high weaning weight was due to the selection of heavier piglets from the commercial farm. It is well known that piglet health closely correlates to body weight after weaning (Tokach et al., 1992) and it is therefore reasonable to assume that the healthy condition of the animals in this study offset possible additional ZnO-effects on their body weight. The relatively low fecal scores observed across all treatment groups also indicate the generally healthy condition of the animals, suggesting that their intestinal function was not adversely affected during the initial phase of the trial.

Nevertheless, significant effects were found as the potentiated ZnO product outperformed the feed-grade ZnO for ADG, ADFI and FCR and thus. Therefore, the physico-chemical properties of ZnO particles are deemed critical to its effectiveness. The effects of potentiated ZnO over standard ZnO were already demonstrated in post weaning piglets (Morales et al., 2012; Wang et al., 2019), where better performance was related to a better intestinal health (Wang et al., 2019) and can be related to a better control of bacterial growth (Vahjen et al., 2012, 2016).

During period 2, the initial ZnO concentration during period 1 of the trial had no effect on ADG or ADFI, but the potentiated ZnO product decreased ADFI. On the other hand, FCR was affected by both ZnO-concentration and -source. Thus, even after withdrawal of higher ZnO concentrations after period 1, persistent effects on FCR were observable. This suggests that there was an improvement in nutrient digestion, which might have started during the initial phase of the trial. As such, the potentiated ZnO product seems to exert a stronger effect than feed-grade ZnO. Overall, ADG and FCR significantly improved for ZnO-concentration and -source; the potentiated ZnO product was again adavantageous compared to the feed-grade ZnO.

Among the hypotheses that may explain the beneficial effect of ZnO in weaned piglets (Lei et al., 2022), physico-chemical properties of ZnO products with increased pore size are discussed to explain differences in bioavailability (Kim et al., 2016) and intestinal solubility (Cardoso, Roméo, et al., 2021).

Furthermore, it has been known for some time that feed-grade ZnO may vary in Zn-concentration (Edwards & Baker, 1999) and consequently, Zn-concentrations have to be aligned in comparative trials. However, varying Zn-concentrations also imply the presence of metal contaminants, potentially including other heavy metals, which can interfere with host physiology. The maximum levels of these contaminants are regulated by Directive 2002/32/EC; however, it’s noteworthy that certain suppliers enforce stricter quality control measures, setting themselves lower maximum allowances for these contaminants. Finally, the intestinal microbiota has been shown to react drastically to pharmacological ZnO-concentrations (Vahjen et al., 2011; Starke et al., 2014) as intestinal bacteria are not only affected by ZnO itself, but also by zinc-modified host physiology.

### Fecal microbiota.

The growth-inhibiting effect of ZnO on Phylum *Firmicutes* could be traced back on the genus level to a decrease in lactobacilli abundance. Lactobacilli are the predominant genus in suckling piglets, but typically decline after weaning due to intake of solid feed (Zhao et al., 2015; Ke et al., 2019). Their high abundance is displaced mostly by members of the *Bacteroidetes* and especially the clostridia, among them the dominating *Clostridium* sensu stricto 1, *Terrisporobacter* and others (Vahjen et al., 2010). This shift from *Lactobacillus* dominance to increased abundance of the clostridia is characteristic for weaned piglets as the microbiota matures into adhulthood (Chen et al., 2017; Guevarra et al., 2019). In this study, increasing zinc concentrations also led to a shift toward higher abundances of bacteria that are predominant in adult animals (for instance *Clostridium* sensu stricto 1, *Terrisporobacter*, genera of *Lachnospiraceae*).

As highlighted in recent literature (Scarpellini et al., 2022), zinc’s effects on the microbiota may be mediated through interactions with the gut mucosa or alterations in digestive physiology. Therefore, the observed shifts in microbial composition and metabolite profiles may partially reflect such indirect effects. Future studies incorporating gut histology and functional markers would help to clarify these interactions more precisely.

However, while the dominating lactobacilli abundance dimished due to higher dietary zinc concentrations, other dominant lactic acid bacteria partly filled the open niche. For instance, streptococci abundance strongly increased with increasing ZnO concentrations. On the other hand, the abundance of bifidobacteria decreased with increasing ZnO concentration until 1500 mg/kg were reached, while the 3000 mg/kg treatment groups both showed strongly increased bifidobacterial abundance. This trend was also visible on the phylum level for the *Proteobacteria* and the *Spirochaetes*. These effects indicate that the bacterial structure in the 3000 mg/kg treatment groups was profoundly altered compared to lower dietary ZnO concentrations, although short chain fatty acid profiles were not different.

Pronounced quantitative and qualitative changes occurred for fecal lactate. Total lactate decreased with increasing ZnO concentrations and this effect correlates directly to the reduced lactobacilli abundance. However, while the L- to D-lactate ratio declined in the feed-grade treatment groups, the potentiated ZnO product showed an increased ratio, indicating a relatively higher L-lactate production by the microbiota. It is known that many bacteria produce D-lactate under oxidative stress or carbon source shortage to detoxify methylglyoxal in a process called “glyoxal shunt” (Maloy & Nunn, 1982; Ferguson et al., 1998; Kosmachevskaya et al., 2015). Thus, changes in the L- to D-lactate ratio can be taken as an indicator for changes in bacterial metabolism and consequently, the feed-grade ZnO may induce additional stress for a considerable part of the fecal microbiota.

It has been shown that pharmacological doses of ZnO increase the number of enterobacteria as well as their diversity (Højberg et al., 2005; Vahjen et al., 2010). An in vitro study also showed that enterobacteria are less affected by ZnO than, for instance, Gram-positive bacteria (Liedtke & Vahjen, 2012). However, the slightly increased amount of enterobacteria and enterobacterial toxin genes in treatment groups fed the potentiated ZnO product may not be alarming for healthy animals, but rather a sign of increased resilience of the animal against possibly detrimental or pathogenic bacteria.

The Linear Discriminant Analysis for Effect Size (LEfSe) finds significant features that most likely explain intergroup differences (Segata et al., 2011). An analysis of all data by source showed that *Lactobacillus*, prevalent in suckling piglets, was the only dominant influential feature in animals fed feed-grade ZnO, while defining features in the potentiated ZnO product were assigned to dominating genera that would be expected in adult animals.

From the above, the authors conclude that the fecal microbiota in animals fed the potentiated ZnO product was advanced in terms of maturity. This may have led to improved gut health, because the intestinal microbiota may have been more efficient in overcoming the unstructured phase after weaning. Incidentally, correlation analysis revealed that the lactobacilli as defining features of the feed-grade ZnO group as well as lactate showed significantly adverse correlations to performance, while *Clostridium* sensu stricto 1 and *Terrisporobacter* showed favorable correlations with the potentiated ZnO product (data not shown). This may be taken as an indication that the maturity of the microbiota indeed correlates to improved performance (Konstantinov et al., 2006; Chen et al., 2017).

Regarding the actual mode of action of dietary ZnO, several hypotheses have been put forward (Bonetti et al., 2021), but the site of action is the intestinal tract and not systemic metabolism (Hahn & Baker, 1993). Solubility issues (Dintzis et al., 1995) and interference from diet components have been addressed (Schlegel et al., 2010), but physico-chemical properties of different ZnO products have to be considered also. Compared to the feed-grade ZnO, the potentiated ZnO product features an enlarged surface area and different particle structure which may play a role in the the observed improvements.

## CONCLUSION

The study demonstrated that piglet performance was closely linked to the levels of dietary zinc oxide, with animals receiving 3000 mg/kg zinc oxide showing superior performance in the initial phase of the experiment compared to those on lower zinc oxide supplemented diets. Long-term impacts on feed efficiency became apparent after stopping the use of high doses of zinc oxide, both in terms of concentration and the source of zinc oxide. When comparing sources, the potentiated zinc oxide variant led to significantly improved feed conversion rates compared to standard feed-grade zinc oxide, suggesting a viable alternative to high pharmacological doses of traditional zinc oxide.

Furthermore, elevating dietary zinc oxide levels led to noticeable changes in fecal microbiota, including a predicted decrease in dominant lactobacilli, an increase in *Proteobacteria*, and a substantially altered bacterial composition at the highest levels of zinc oxide supplementation. In general, the potentiated zinc oxide product appeared to support the growth of a more mature microbiota.

## Supplementary Material

txaf073_suppl_Supplementary_Tables_S1-S4_Figure_S1

## Data Availability

The 16S rDNA sequencing data generated and analyzed during this study have been deposited in the NCBI Sequence Read Archive (SRA). The data can be accessed using the accession number [PRJNA1261819].
