# Supplementary material for: Effects of different doses and preparations of zinc oxide in weanling piglets on performance, intestinal microbiota and microbial metabolites
Source: Transl Anim Sci. 2025 Jun 20;9:txaf073. doi: 10.1093/tas/txaf073 (PMC12260153; doi:10.1093/tas/txaf073)
Supplement: txaf073_suppl_Supplementary_Tables_S1-S4_Figure_S1 [file txaf073_suppl_supplementary_tables_s1-s4_figure_s1.docx]

# Supplemental Information

**Supplemental Table 1: Analyzed nutrients and mineral concentrations of the starter and grower diets used in the trial**

| Key proximate analysis and trace mineral contents of experimental starter diets (as is) | | | | | | | | | | | | | |
| --- | --- | --- | --- | --- | --- | --- | --- | --- | --- | --- | --- | --- | --- |
| **Items/Treatment group** |  | **Z150** | **Z300** | **Z600** | **Z900** | **Z1500** | **Z3000** | **H150** | **H300** | **H600** | **H900** | **H1500** | **H3000** |
| Dry matter | g/kg | 898.2 | 898.9 | 898.6 | 899.0 | 898.5 | 898.7 | 899.2 | 899.0 | 898.7 | 898.3 | 898.4 | 897.9 |
| Crude protein | g/kg | 199.0 | 200.3 | 197.2 | 198.7 | 198.6 | 199.4 | 196.1 | 200.1 | 200.3 | 199.2 | 198.9 | 199.1 |
| Crude fibre | g/kg | 39.86 | 38.84 | 38.45 | 38.08 | 37.42 | 38.51 | 37.96 | 36.41 | 36.86 | 37.99 | 37.78 | 38.11 |
| Crude fat | g/kg | 20.10 | 20.01 | 19.94 | 20.07 | 20.48 | 20.45 | 20.56 | 20.32 | 19.63 | 19.73 | 20.59 | 20.62 |
| Ash | g/kg | 59.14 | 57.10 | 58.12 | 58.26 | 54.02 | 53.18 | 58.96 | 57.05 | 57.82 | 57.42 | 56.52 | 53.08 |
| Starch | g/kg | 404.3 | 407.4 | 408.0 | 402.5 | 399.3 | 404.3 | 403.1 | 407.1 | 398.3 | 404.3 | 398.7 | 399.6 |
| Iron | g/kg | 290.16 | 313.25 | 325.96 | 326.65 | 328.23 | 324.07 | 287.07 | 312.45 | 304.14 | 320.71 | 298.27 | 279.74 |
| Manganese | g/kg | 102.19 | 107.15 | 101.25 | 98.79 | 97.16 | 99.89 | 101.08 | 102.8 | 97.19 | 95.86 | 97.36 | 98.15 |
| Zinc | g/kg | 137.7 | 224.3 | 544.5 | 661.6 | 1069.6 | 1985.7 | 150.9 | 203.8 | 536.5 | 679.8 | 1089.5 | 2042.0 |
| Copper | g/kg | 20.11 | 19.27 | 18.57 | 19.46 | 20.30 | 20.05 | 19.96 | 20.98 | 19.69 | 18.05 | 20.16 | 19.28 |
| Key proximate analysis and trace mineral contents of experimental grower diets (as is) | | | | | | | | | | | | | |
| **Items/Treatment group** |  | **Z150** | **Z300** | **Z600** | **Z900** | **Z1500** | **Z3000** | **H150** | **H300** | **H600** | **H900** | **H1500** | **H3000** |
| Dry matter | g/kg | 895.9 | 896.1 | 897.0 | 896.7 | 897.3 | 898.4 | 897.2 | 898.1 | 897.6 | 897.4 | 896.9 | 896.3 |
| Crude protein | g/kg | 186.5 | 184.3 | 184.9 | 185.3 | 187.1 | 183.9 | 184.6 | 189.5 | 187.6 | 188.4 | 187.0 | 187.2 |
| Crude fibre | g/kg | 32.68 | 30.4 | 33.29 | 31.93 | 32.45 | 32.72 | 31.85 | 31.07 | 32.92 | 32.56 | 32.08 | 31.55 |
| Crude fat | g/kg | 22.14 | 21.47 | 20.99 | 21.67 | 22.90 | 21.93 | 21.85 | 22.70 | 21.98 | 21.48 | 21.60 | 21.87 |
| Ash | g/kg | 52.44 | 51.16 | 51.67 | 50.74 | 51.16 | 50.81 | 50.45 | 53.68 | 53.81 | 52.81 | 53.89 | 51.40 |
| Starch | g/kg | 419.7 | 421.7 | 421.7 | 418.4 | 419.1 | 414.8 | 416.1 | 417.4 | 418.7 | 420.4 | 419.1 | 420.3 |
| Iron | g/kg | 219.3 | 220.53 | 205.02 | 222.32 | 223.5 | 219.84 | 219.00 | 216.29 | 220.69 | 215.87 | 212.56 | 214.04 |
| Manganese | g/kg | 90.67 | 84.83 | 85.95 | 82.90 | 82.73 | 85.07 | 84.00 | 83.7 | 85.25 | 85.83 | 86.21 | 84.83 |
| Zinc | g/kg | 159.0 | 166.3 | 150.1 | 149.7 | 148.8 | 155.6 | 146.2 | 150.5 | 149.7 | 153.7 | 153.7 | 155.3 |
| Copper | g/kg | 18.05 | 18.65 | 18.77 | 18.76 | 17.87 | 18.60 | 17.95 | 18.39 | 17.93 | 18.01 | 17.84 | 18.03 |

**Supplemental Table 2: Chemical composition and physicochemical characteristics of the two ZnO sources used in the trial**

|  | ZnO Maximo | HiZox^®^ |
| --- | --- | --- |
| Zn content, % | 72 | 76 |
| Lead, mg/kg of product | 120 | 14 |
| Cadmium, mg/kg of product | 1.2 | 0.3 |
| Arsenic, mg/kg of product | 1.8 | 0.1 |
| Iron, mg/kg of product | 36,000 | 19 |
| Particle size, µm | 44 | 126 |
| Specific surface area, m^2^/g | 0.3 | 45 |
| Density, g/mL | 2.5 | 1 |
| Speed of dissolution kinetics at pH 4 | Very slow | Medium |
| Type of ZnO | Large Dense Particles | Small Aggregated & Agglomerated Particles |

**Supplemental Table 3: Primer and PCR conditions**

| Primer name | Annealing- temp. | Forward | Reverse | Literatur |
| --- | --- | --- | --- | --- |
| *Entero* | 55°C | GTTAATACCTTTGCTCATTGA | ACCAGGGTATCTAATCCTGTT | Malinen, E. Kassinen, A. Rinttilä, T. and Palva, A. (2003). Comparison of real-time PCR with SYBR Green I or 5'-nuclease assays and dot-blot hybridization with rDNA-targeted oligonucleotide probes in quantification of selected fecal bacteria Microbiology 149, 269-277. |
| *estIb* | 55°C | CAACTGAATCACTTGACTCTT | TTAATAACATCCAGCACAGG | Bosworth, B.T. and Casey, T.A. (1997). Identification of toxin and pilus genes in porcine E. coli using polymerase chain reaction (PCR) with multiple primer pairs. Proceedings of the 97th Annual General Meeting, abstract B-509. Am. Soc. Microbiol., Miami, Florida |
| *estII* | 55°C | TGCCTATGCATCTACACAAT | CTCCAGCAGTACCATCTCTA | Bosworth, B.T. and Casey, T.A. (1997). Identification of toxin and pilus genes in porcine E. coli using polymerase chain reaction (PCR) with multiple primer pairs. Proceedings of the 97th Annual General Meeting, abstract B-509. Am. Soc. Microbiol., Miami, Florida |
| *FedA* | 55°C | TGGTAACGTATCAGCAACTA | ACTTACAGTGCTATTCGACG | Bosworth, B.T. and Casey, T.A. (1997). Identification of toxin and pilus genes in porcine E. coli using polymerase chain reaction (PCR) with multiple primer pairs. Proceedings of the 97th Annual General Meeting, abstract B-509. Am. Soc. Microbiol., Miami, Florida |
| *fae* | 63°C | GCACATGCCTGGATGACTGGTG | CGTCCGCAGAAGTAACCCCACCT | Bosworth, B.T. and Casey, T.A. (1997). Identification of toxin and pilus genes in porcine E. coli using polymerase chain reaction (PCR) with multiple primer pairs. Proceedings of the 97th Annual General Meeting, abstract B-509. Am. Soc. Microbiol., Miami, Florida |

| **Supplemental Table 4: Effect of two different dietary sources of zinc oxide (feed-grade ZnO and a potentiated ZnO product) on fecal concentrations of lactic acid and short chain fatty acid in weaned piglets on day 14 of the trial** | | | | | | | | | | | | |
| --- | --- | --- | --- | --- | --- | --- | --- | --- | --- | --- | --- | --- |
|  | | | | | | | | | | | | |
|  |  |  |  |  |  |  |  |  |  |  | P-value^2^ |  |
|  | Z150 | Z600 | Z1500 | Z3000 | H150 | H600 | H1500 | H3000 | SEM^1^ | C | S | CxS |
|  | | | | | | | | | | | | |
| Lactic acid, µmol/g |  |  |  |  |  |  |  |  |  |  |  |  |
| L-Lactic acid | 2.58 | 0.99 | 0.13 | 0.42 | 1.93 | 2.19 | 0.49 | 0.37 | 0.338 | < 0.001 | 0.446 | 0.128 |
| D-Lactic acid | 0.38 | 0.90 | 0.30 | 0.36 | 1.10 | 1.28 | 0.15 | 0.10 | 0.161 | < 0.001 | 0.297 | 0.120 |
| Total lactic acid | 2.97 | 1.89 | 0.43 | 0.78 | 3.04 | 3.47 | 0.64 | 0.47 | 0.456 | < 0.001 | 0.369 | 0.427 |
| L/D Lactate ratio | 5.55 | 1.05 | 0.39 | 0.70 | 1.16 | 2.13 | 4.17 | 7.76 | 0.288 | 0.014 | < 0.001 | < 0.001 |
| Short chain fatty acids, µmol/g |  |  |  |  |  |  |  |  |  |  |  |  |
| Acetic acid | 70.0 | 70.0 | 68.8 | 82.4 | 79.8 | 82.5 | 73.0 | 72.2 | 2.044 | 0.313 | 0.115 | 0.011 |
| Propionic acid | 26.9 | 26.0 | 25.6 | 28.6 | 27.5 | 27.7 | 26.8 | 27.9 | 0.350 | 0.494 | 0.500 | 0.830 |
| Isobutyric acid | 2.86 | 2.76 | 2.81 | 2.91 | 2.78 | 3.23 | 3.01 | 2.35 | 0.088 | 0.137 | 0.928 | 0.013 |
| Butyric acid | 19.1 | 16.7 | 16.5 | 18.7 | 18.6 | 16.4 | 17.8 | 17.1 | 0.382 | 0.244 | 0.733 | 0.655 |
| Isovaleric acid | 5.18 | 4.66 | 4.79 | 4.69 | 4.40 | 5.03 | 5.05 | 3.73 | 0.164 | 0.037 | 0.146 | 0.024 |
| Valeric acid | 4.25 | 3.97 | 3.85 | 3.95 | 3.95 | 4.02 | 3.96 | 3.53 | 0.071 | 0.534 | 0.428 | 0.675 |
| Total SCFA | 128 | 124 | 122 | 141 | 137 | 139 | 130 | 127 | 2.509 | 0.539 | 0.334 | 0.074 |
| Branched fatty acids, µmol/g |  |  |  |  |  |  |  |  |  |  |  |  |
| Total BCFA | 8.04 | 7.41 | 7.60 | 7.60 | 7.18 | 8.26 | 8.06 | 6.08 | 0.243 | 0.065 | 0.373 | 0.020 |
|  | | | | | | | | | | | | |

^1^Standard error of the means

^2^P-values for the main and interactive effects of the concentration (C) and source (S) of zinc

**Supplemental Figure 1: Effect of two different dietary sources of zinc oxide (feed-grade ZnO and a potentiated ZnO product) on dominant bacterial genera in weaned piglets on day 14 of the trial**
